# Physio-Chemical and Biological Characterization of Novel HPC (Hydroxypropylcellulose):HAP (Hydroxyapatite):PLA (Poly Lactic Acid) Electrospun Nanofibers as Implantable Material for Bone Regenerative Application

**DOI:** 10.3390/polym15010155

**Published:** 2022-12-29

**Authors:** S. Mary Stella, T. M. Sridhar, R. Ramprasath, Jolius Gimbun, U. Vijayalakshmi

**Affiliations:** 1 Department of Chemistry, School of Advanced Sciences, Vellore Institute of Technology, Vellore 632014, India; 2 Department of Analytical Chemistry, Guindy Campus, University of Madras, Chennai 600025, India; 3 Department of Chemical Engineering, College of Engineering, Universiti Malaysia Pahang, Pekan 26600, Malaysia

**Keywords:** electrospinning, polymer, composite, XPS (X-ray photoelectron spectroscopy), tensile properties, in vitro study

## Abstract

The research on extracellular matrix (ECM) is new and developing area that covers cell proliferation and differentiation and ensures improved cell viability for different biomedical applications. Extracellular matrix not only maintains biological functions but also exhibits properties such as tuned or natural material degradation within a given time period, active cell binding and cellular uptake for tissue engineering applications. The principal objective of this study is classified into two categories. The first phase is optimization of various electrospinning parameters with different concentrations of HAP-HPC/PLA(hydroxyapatite-hydroxypropylcellulose/poly lactic acid). The second phase is in vitro biological evaluation of the optimized mat using MTT (3-(4,5-dimethylthiazol-2-yl)-2,5-diphenyl tetrazolium bromide) assay for bone regeneration applications. Conductivity and dielectric constant were optimized for the production of thin fiber and bead free nanofibrous mat. With this optimization, the mechanical strength of all compositions was found to be enhanced, of which the ratio of 70:30 hit a maximum of 9.53 MPa (megapascal). Cytotoxicity analysis was completed for all the compositions on MG63 cell lines for various durations and showed maximum cell viability on 70:30 composition for more than 48 hrs. Hence, this investigation concludes that the optimized nanofibrous mat can be deployed as an ideal material for bone regenerative applications. In vivo study confirms the HAP-HPC-PLA sample shows more cells and bone formation at 8 weeks than 4 weeks.

## 1. Introduction

Bone tissue engineering is one of the most important approaches to repairing damaged and diseased tissue and it facilitates the complete recovery of the tissue itself as well as its function [1]. Bone serves several functions such as body support, organ protection, and storage of nutrients. Due to the applicability of bone, it is considered a complex tissue in the human body [2]. Moreover, it has an extremely anisotropic nature due to a variety of mechanical properties extending in all directions. The design of scaffolds as an extracellular matrix (ECM) and the process of regeneration is the major goals in tissue engineering [3]. ECM should be nontoxic and biocompatible as well as show the desired degradation rate and porosity with excellent mechanical properties. Additionally, ECM should not be the cause of foreign body reactions [4].

Alternatively, scaffolds are used as supporting materials and the results have been better cell growth, increased proliferation rate, and healthier ECM production [5]. The fabricated artificial material will be useful for the repair of damaged tissue and the regeneration of new tissues by cell proliferation and growth of their own ECM. Generally, several types of material sources, be it natural, synthetic, semi-synthetic or even composites are used to synthesize scaffolds used in bone tissue regeneration.

Cellulose fiber is a good choice for bone tissue engineering due to its high strength and better mechanical properties. Subsequently, the fabrication of cellulose composite scaffolds is attracting many researchers due to its highly applicable mechanical properties [6]. The main advantages of cellulose-based scaffolds are biodegradability in chemical as well as biological environments and mechanical stress factors, good mechanical properties in terms of tensile strength, negligible foreign body and inflammatory response reactivity within in vitro and in vivo applications, and abundant presence of this naturally occurring polymer [7,8]. In tissue engineering, cellulose has been used as a permanently implantable scaffold establishing the fact that it is stable for a long-time application in vivo [9,10].

Among the other cellulose fibers, hydroxypropyl cellulose (HPC) has the above-mentioned desired properties and it can be used for the fabrication of HAP-reinforced fibrous mat. Structurally, hydroxypropyl groups substitute the hydroxyl groups present in cellulose, hence yielding better mechanical properties than cellulose [11]. The material has adequate biomedical application due to its biodegradability and biocompatibility [12].

In scaffold fabrication, polymers play a major role as they are responsible to mimic the organic nature of human native bone. Poly(lactic acid) (PLA) [13], polyglycolic acid (PGA) [14], poly(lactic-co-glycolic acid) (PLGA) [15], poly(vinyl alcohol) (PVA) [16], poly(vinyl pyrrolidone) (PVP) [17], poly(methyl methacrylate) [18] and polycaprolactone (PCL) [19] are some of the important polymers that may be deployed in fabricating scaffolds for bone tissue engineering applications. Among the above polymers, PLA has advantages such as biocompatibility, biodegradability and good modulus with adequate strength [20].

Certain polymeric materials are unable to accomplish the desired scaffold to provide valuable inputs in biomedical applications. To overcome these issues, the composite of natural and synthetic polymers can be employed. HPC polymer has good biocompatibility and fine particle grades are most favorable for faster hydration, and uniform dissolution of particles during mixing [21]. On the other hand, it has some disadvantages such as hydrophobicity and low molecular weight. Additionally, for electrospinning to occur, polymer chain entanglement is one of the important criteria to form nanofibers. Due to these reasons, HPC as such is not spinnable and hence the polymers such as PLA are mixed to provide sufficient chain entanglement in the solution, whereby electro-spinnability is feasible [22].

Hydroxyapatite [Ca_10_(PO_4_)_6_(OH)_2_] is one of the important materials which offer significant applications in bone therapy and tooth reconstruction. It exhibits the properties and mechanisms to directly bond to the natural host tissue and results in regeneration [23]. The major challenge in recent research is producing an apatite layer similar to human tissues to be confirmed by morphological and physiochemical analysis. It is necessary to understand the mechanism of HAP bonding and the biomineralization process leading to cell growth [24]. In biomedical aspects, scaffolds have been fabricated to renew or repair damaged organs by fracture or defects caused by disease (osteoporosis, osteoarthritis, osteosarcoma) or surgeries (tumor removal) or congenital disabilities. To figure out, the risk and demands, artificial scaffolds have been fabricated with the properties of biocompatibility, biodegradability, mineralization, mechanical resistance, and cell proliferation.

Electrospinning is an emerging technique for the fabrication of nanofibrous scaffolds which is useful for the production of ECM better than other bone scaffold fabrication techniques [25]. As we know, other fabrication techniques do not offer any ability to control porosity, whereas the electrospinning technique offers continuous fiber production with a controlled diameter. The diameter and alignment of the fiber can be easily controlled by adjusting the properties of the HAP–polymer composite solution and parameters of the electrospinning instrument [26].

In the present study, the novelty of the work is divided into two categories: Firstly, HAP-HPC/PLA nanofibrous mat has been fabricated using electrospinning techniques. The composite of HAP-HPC/PLA prepared by 25% of HAP was gradually added into 5 wt% of HPC/PLA polymer solution. The different composition ratios such as 0:100, 40:60, 50:50, 60.40, and 70:30 of HAP-HPC/PLA have been used to optimize the composition in terms of mechanical strength, biodegradability, and bioactivity properties. Different physical, chemical, and mechanical characterization techniques were used for the analysis of composite nanofibers. Further, the optimized scaffold has been confirmed by in vitro biocompatibility study. In this study, the cytotoxicity analysis has been carried out using the MG63 (osteoblast) cell line to achieve the osteoconductive property in the form of new bone formation. Secondly, the optimized composite was further confirmed by in vivo performance using the calvarial defect on rats and qualitative analysis was carried out at 4 weeks and 8 weeks of implantation using radiological (X-Ray) evaluation and histological study. The objective of this work was to optimize composite scaffolds with respect to the different levels of cell growth (osteoblast and osteoclast etc.). Thus, the optimized study plays a favorable foundation for new bone formation both by in vitro and in vivo analysis.

## 2. Materials and Methods

### 2.1. Materials

PLA (molecular weight of 220 kDa), HPC (molecular weight of 100,000 kDa), aqueous ammonia, calcium nitrate tetrahydrate (Ca(NO_3_)4H_2_O) and ammonium dihydrogen orthophosphate (NH_4_H_2_PO_4_) were purchased from the SD-Fine chemicals, Mumbai, India.

MG63 cell lines were obtained from NCCS, Pune. DMEM (Dulbecco’s Modified Eagle Medium), fetal bovine serum (FBS), trypsin and 1× antibiotic solution were purchased from Hi-Media Laboratories, Mumbai, India. Methyl thiazolyl diphenyl-tetrazolium bromide (MTT) and dimethyl sulfoxide (DMSO) were purchased from Sigma-Aldrich Chemicals Company, Mumbai, India. The cells were maintained in Dulbecco’s Modified Eagle’s Medium (DMEM) supplemented with 10% FBS, penicillin (10,000 U/mL), and streptomycin (10 mg/mL) in a humidified atmosphere of 50 μg/mL CO_2_ at 37 °C.

### 2.2. Methods

#### 2.2.1. Preparation of Hydroxyapatite and Polymer Solution

1M solutions of calcium nitrate tetrahydrate and 0.6M of ammonium dihydrogen orthophosphate were prepared using double distilled water by adopting co-precipitation method. Both the solutions were mixed together under continuous stirring and pH of the solution was adjusted to 11 by adding aqueous ammonia [27]. Pure hydroxyapatite was obtained by aging the solution for 16 h, the raw powder was filtered and washed several times with distilled water followed by sintering the dry powder at 700 °C for 2 h. Nanofibrous scaffold was fabricated using 2.5 g of HAP powder dispersed in 10 mL of water under stirring conditions for 12 h. The solution of HPC/PLA was prepared by selecting 0.5 g of (5 wt %) HPC and 0.5 g of PLA in 10 mL of boiling water under stirring conditions. Finally, the resultant solution with the composition of HAP/HPC/PLA suspension was made for electrospinning.

The composite mixture was stirred for 3 h until a clear homogeneous solution was obtained. The homogenous solution is allowed to age for 12 h followed by ultra-sonication prior to electrospinning.

#### 2.2.2. Viscosity and Electrical Conductivity Analysis

The obtained composite solutions were tested for their viscosity using Brookfield DV2 viscometer. The dielectric constant of the electrospun nanofibers was measured using a HIOKI 3532-50 LCR HITESTER meter at a temperature range of 35–125 °C at a frequency of 0–5 × 106 Hz.

#### 2.2.3. Electrospinning

The nanofibrous mat was fabricated with different composite ratios (0:100, 40:60, 50:50, 60:40, and 70:30) of HAP and HPC/PLA. The composite mixture was stirred for 3 h until a clear homogeneous solution is obtained. The homogenous solution is allowed to age for 12 h. The composite solution to be electrospun was taken in a plastic syringe (5 mL) with a hypodermic needle and a flat-filed tip, with an internal diameter of 0.8 mm. The electrospun nanofibers were collected on aluminum foils on a rotating drum collector at a controlled relative humidity (20–25%) environment. The obtained nanofibrous mat was dried along with aluminum foil in a desiccator to reduce the effect of humidity on the nanofibers [28]. The solution preparation is tabulated in Table 1. 

#### 2.2.4. Physio Chemical Characterization

FT-IR analysis was used to distinguish species, and analyze the functional groups, vibration modes, chemical interaction of HAP with polymers. Evaluation by using FT-IR was accomplished using SHIMADZU CROP IRAFFINITY-1 flourier transform infrared spectrophotometer within the variety 400–4000. X-ray diffraction is used to identify the nanoparticles such as HAP in composites. This characterization was performed for dried and finely tailored nano composite samples on XRD machine (BRUKER Germany with Cu K radiation; =1.5405 Å). Mechanical study used to measure the tensile strength and elastic modulus. Tensile strength for electrospun nanofibers was performed using an ASTM standard (D695) on the Tinus Olsen H5K5 universal testing machine. Nanofibers were cut into a rectangular shape of width 12 mm and placed at the height of 6 cm between two clamps bearing a 500 N load cell with velocity of 1mm/min. The average of three trials of tensile modulus were calculated from stress–strain response. X-ray photoelectron spectroscopy (XPS, ESCALAB 250Xi, Richardson, TX, USA) is used to analyze materials surface chemistry. Specimens were analyzed using a monochromatic Al Kα source (10 mA, 15 kV). Scanning Electron Microscope (ZISS-EVO18, Horn, Austria) is used to investigate the morphology of composite at excessive magnification and resolution by using lively electron beam.

#### 2.2.5. Porosity

The solvent replacement method was used for the measurement of porosity of the HPC/PLA and HAP-HPC/PLA nanofibrous mat. The initial weight (Wi) of the nanofibers was measured after drying in desiccator. The nanofibrous mat was immersed in absolute ethanol and dried off immediately. The porosity of the electrospun nanofibrous mat was determined as follows:

Porosity = ((Ws − Wi))/Ws × 100(1)

where Ws is the rehydrated nanofiber weight and Wi is the initial nanofiber weight.

#### 2.2.6. Bioactivity Study

##### Bioactivity

The simulated body fluid (SBF) solution which is similar to the human physiological fluid was used for the in vitro bioactive study of the electrospun nanofibers. The procedure, which was suggested by Kokubo et al., 1990 [29] was used for the preparation of SBF solution. All chemicals were added together to 1 L of double distilled water, pH was adjusted to 7.35 ± 0.25, and stored in the refrigerator at −4 °C. For the analysis, 1 × 1 cm size of the electrospun nanofibrous mat (HPC/PLA and HAP-HPC/PLA) was immersed in the SBF solution (60 mL) at 37 °C for a different time duration of 7, 15, and 30 days, respectively. The contamination was avoided by the replacement of SBF solution with fresh solution for every 24 h. Carbonated hydroxyapatite (HCA) was formed in the process of immersion in the SBF solution and hence resulting in the formation of apatite layer. SEM and EDAX analysis were used for the confirmation of apatite layer formation on the surface of the electrospun nanofibers.

##### In Vitro Biodegradation Properties

Electrospun mat of dimensions 1 × 1 cm was immersed in SBF solution at a pH of 7.4 for different time intervals such as 7, 15, and 30 days, respectively. The samples were dried at room temperature after the respective time duration was completed [30]. The percentage degradation was calculated from the dried weight of the samples after degradation as follows:

Percentage Degradation = ((Wi − Wd))/Wi × 100(2)

where Wi and Wd are initial and dried weight of the sample, respectively.

#### 2.2.7. Cytotoxicity Analysis

Osteosarcoma (MG-63) cell lines were used for the cytotoxicity analysis of the fabricated mat. MG63 was passaged using Minimum Eagle’s Medium (MEM), 10% fetal bovine serum, and 1% penicillin–streptomycin solution and maintained at 37 °C and 5% CO_2_ concentration in a humidified incubator. Mats were sterilized by washing with 70% ethanol 2–3 times and exposing them to UV overnight (approximately for 12 h). The sterilized sample was soaked in cell culture medium for 2 h. The cells were harvested using 0.05% (*v/v*) trypsin–EDTA (ethylenediamine tetraacetic acid) and seeded on scaffolds followed by incubation at 37 °C in the presence of CO_2_ environment. The medium was changed once in 2 days to supply the adequate amount of nutrients present in the culture plate. Further, 3-[4,5-dimethylthiazol-2-yl]-2,5-diphenyltetrazolium bromide (MTT) assay was used to check the toxicity effects of samples. The media were washed off with 0.1 M PBS solution at the end of 24 and 48 h incubation. Further, 300 µL of fresh media and 60 µL of MTT solution were mixed together, added to PBS-washed mats, and incubated at 37 °C for 3 h. About 200 µL of the incubated mixture was filled in 96-well plate and absorbance was measured at 570 nm using a Vmax Microplate reader. Triplicates were used for calculating the average of two sets of the assay [31]. The % cell viability was calculated using the following formula:

%cell viability ((A570 of treated cells))/((570 of control cells)) × 100(3)

#### 2.2.8. Statistical Analysis

Results were expressed as a mean and standard deviation. Comparative studies of means were performed using one-way analysis of variance (ANOVA). Significance was accepted with *p* < 0.05.

#### 2.2.9. In Vivo Study

Sixteen 4–6 weeks-old Wistar albino rats weighing between 250 and 400 g were obtained from the Animal Center Laboratory of VIT University. All experimental rats were bred at the Animal Center Laboratory of VIT University, with a standard laboratory diet and environment. All animal experiments were approved and performed according to the regulations of the animal ethics committee of our university. VIT/IAEC/14/NOV5/47. The rats were anesthetized by intraperitoneal injection of pentobarbital (ketamine 0.2 mL and xylene 0.1 mL). Using sterile instruments and aseptic technique, a 1.0–1.5 cm sagittal incision was made on the scalp, and the calvarium was exposed by blunt dissection. A full-thickness defect (5 mm in diameter) was created in the central area of each parietal bone using a 5 mm electric trephine bur under constant irrigation with sterile 0.9% saline. The defects were implanted randomly with the HAP-HPC-PLA scaffolds (n ¼ 12). After 4 and 8 weeks of implantation, the calvaria of the rats was harvested and immediately immersed in a 10% tempered solution of formalin and further analysis was studied such as R-ray and histopathological.

## 3. Results and Discussion

### 3.1. Preparation of HAP-HPC/PLA Nanofibers

The solution of HPC/PLA was prepared by HPC and was added to boiling water. Since HPC has a solubility issue, PLA with high viscosity was added to HPC to make the solution suitable for electrospinning. The different amounts of HAP-HPC/PLA were considered to fabricate nanofibers of different compositions.

### 3.2. Viscosity, Electrical Conductivity, and Dielectric Constant Analysis

HAP was blended with HPC/PLA polymer composite at different ratios such as 0:100 (HAP is absent here), 40:60, 50:50, 60:40, and 70:30 and these resulted in a viscosity of 387.6 2487, 1413, 1198, and 786.7 cP, respectively. Amongst the different composite solutions, 70:30 mixture had a viscosity of 786.7 cP, which is found to be the applicable viscosity, as it has to flow easily from the needle during electrospinning. Additionally, the viscosity parameter is significant, because the optimum viscosity can only yield a targeting diameter of nanofibers for various applications. The viscosity and conductivity of HAP-HPC/PLA composite solutions were measured and mentioned in Table 2.

**Figure 1:**
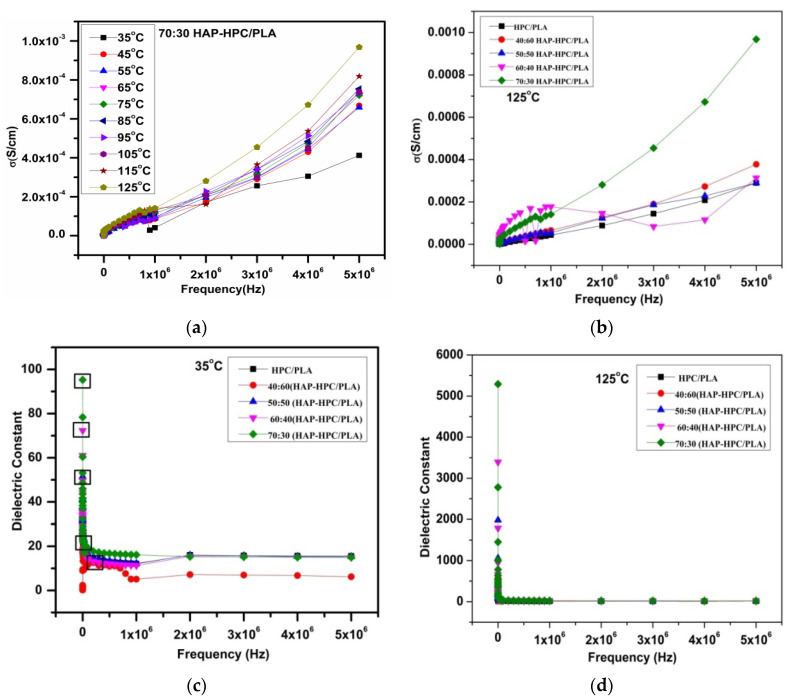
(**a**) Conductivity of HAP-HPC/PLA (70:30); (**b**) different ratios of HAP-HPC/PLA; (**c**) dielectric constant at 30 °C of 0% HAP and HAP-HPC/PLA; (**d**) dielectric constant at 125 °C of 0% HAP and HAP-HPC/PLA.

Conductivity and dielectric constants depend on the input temperature, frequencies, and viscosity of the solution. The overall parameters and conductivity depend on the viscosity of the sample preparation. Voltage, RPM (rotations per minute), and conductivity are the main parameters to form thinner fiber diameters. Conductivity remains constant at low temperatures, and it increases only when the temperature is increased. The dielectric constant and conductivity study of the nanofiber scaffolds is shown in Figure 1. In this study, the conductivity (σ) and dielectric constants (ε) depend on the concentration of the HAP at different temperature and the frequency was studied. The conductivity (σ) and dielectric constant (ε) were found to be increasing with an increase in temperature (35 to 125 °C) and with an increase in HAP concentration.

The dielectric constant decreases with increasing frequency and we obtain the maximum value of the dielectric constant 96 K for a 70:30 ratio of the nanofibrous mat at 5 kHz at room temperature. This is because of the fact that at lower frequency dipole moment of hydroxyl ions in HAP follow the variation of the field while at higher frequency those ions do not follow the variation of the field and the dielectric permittivity value decreases. Figure 1b shows the frequency dependence of the dielectric constant for different compositions at room temperature and at 125 °C. The dielectric constant increases with increasing the HAP content and we obtain the value of dielectric constant 5329 K at 5 kHz at 125 °C and at 89 K for RT which is due to the interfacial flow rate of the viscous solution during electrospinning. Similarly, the conductivity has a much stronger concentration dependence with respect to frequency. Hence, from the results observed, the values of both conductivity and dielectric constant for the 70:30 ratio were found to be increased in HAP-HPC/PLA composition. The percolation threshold limit of 70:30 ratio of HAP-HPC/PLA is flexible and hence applicable for the fabrication of mat.

### 3.3. FT-IR (Fourier Transform Infrared) Analysis of HAP-HPC/PLA Nanofibers

**Figure 2:**
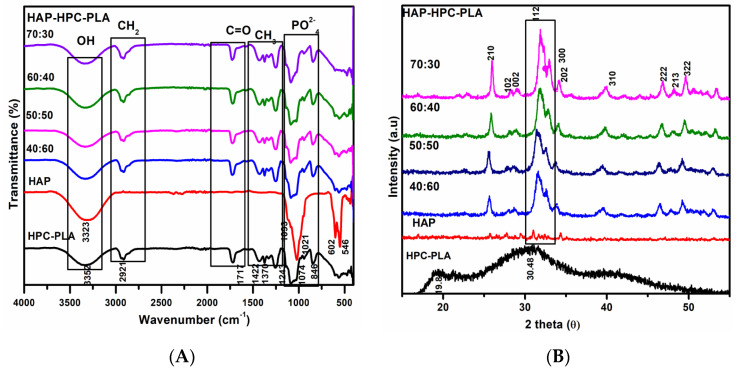
(**A**) FTIR and 2 (**B**) XRD (X-ray diffraction) analysis of HAP-HPC/PLA nanofibers of different ratios.

The FT-IR spectra of the fabricated HPC/PLA nanofibrous mat and HAP with various composite ratios of 40:60, 50:50, 60:40, and 70:30 have been illustrated in Figure 2A. The absorption band at 3351 cm^−1^ corresponds to a hydroxyl group in the pyranose unit of HPC. The absorption band at 2921 cm^−1^ appears due to the CH2 and CH stretching vibration. The absorption peaks at 1717 and 1422 cm^−1^ are attributed to the C=O stretch and –CH stretch, respectively. The absorption band appearing at 1079 cm^−1^ is formed due to C-O stretching vibration. The results confirm the presence of HPC in the composite nanofibrous mat.

Figure 2A shows the broad peak at 3344 cm^−1^ corresponding to the OH stretch. Mainly, the prominent peaks appearing at 478 cm^−1^, 555 cm^−1^, 936 cm^−1^, 1021 cm^−1^, and 1081 cm^−1^ are characteristic bands assigned to PO_4_^3−^ of HAP. The distinguishable peak at 936 cm^−1^ appears due to the asymmetric P-O stretching vibration of PO_4_^3-^ bands. The sharp peaks at 478 cm^−1^ and 555 cm^−1^ correspond to the triply degenerate bending vibrations of PO_4_^3−^ in HAP and these results further confirm the presence of HAP. Additionally, the characteristics peaks of HPC/PLA appear at 2931 cm^−1^ and 1437 cm^−1^ responsible for the asymmetric CH_2_ and CH_3_ stretch. The prominent peak at 1756 cm^−1^ identifies the carbonyl group. Eventually, FTIR spectroscopy results of Figure 2A confirmed that fabricated nanofibers contain HAP and HAP-PLA in all composite nanofibrous mats.

### 3.4. XRD Analysis of HAP-HPC/PLA Nanofibers

The phase analysis and purity of the fabricated HAP-HPC/PLA nanofibrous composite mats have been scanned using XRD studies which are shown in Figure 2B. The diffraction peaks at 19.8° and 30.48° are responsible for the HPC/PLA composite. Additionally, the diffraction peaks of HAP in HAP-HPC/PLA nanofibrous composite mats at 22.78°, 25.92°, 28.10°, 29.12°, 31.89°, 32.05°, 32.20°, 34.09°, 39.88°, 46.75°, 48.28°, 49.48°, 50.78°, and 51.23° are corresponding to (111), (002), (102), (210), (211), (112), (300), (202), (310), (222), (312), (213), (321), and (410) planes are confirmed. Hence both FTIR and XRD results confirmed the presence of pure HAP without any other secondary phases. Moreover, the triplet peak appearing at 31.89°, 32.05°, and 32.20° is due to an increase in the HAP concentration in the fabricated mats. The results suggest that the diffraction peak of HPC/PLA (Figure 2B) is slightly amorphous. Further, the intensity of the diffraction peaks is increasing with the addition of HAP to the HPC/PLA composite.

### 3.5. Mechanical Properties

**Figure 3:**
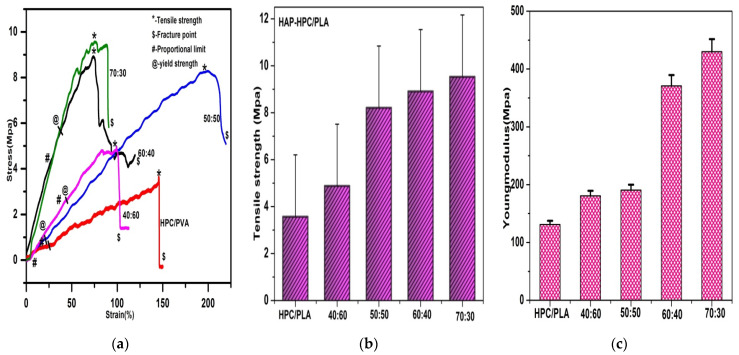
Correlation of (**a**) mechanical property (**b**) tensile strength (**c**) Young modulus.

As explained above, the smaller diameter of fibers with a definite orientation of the polymer chains has been observed in this study. The extended amorphous structures of the HPC/PLA and HAP-HPC/PLA nanofibers yield results as shown in Figure 3. The obtained results suggest that 70:30 ratio shows moderate tensile strength compared to the other ratios (Figure 3a). The results demonstrated that tensile strength and Young’s modulus are found to be increased due to the alignment of the polymer along the axes of fibers, although the percentage of fracture is found to be decreasing, respectively. HAP-HPC/PLA nanofibrous mat with a composition ratio of 70:30 resulted in the high tensile strength of 9.53 MPa. The other composite ratios of 0:100, 40:60, 50:50, and 60:40 gave a tensile strength of 3.7 MPa, 4.88 MPa, 8.21 MPa, and 8.91 MPa, respectively. Further, Table 3 shows an increase in tensile strength as the percentage of HAP increases; hence maximum tensile strength was observed for “70:30” composition of HAP-HPC/PLA nanofibrous mat. Additionally, the percentage of fracture has decreased, and Young’s modulus has increased, exhibiting high stiffness, regular chain orientation, and elevated mechanical strength to the nanofibers. The composite mat without HAP shows less Young’s modulus and tensile strength. All these results clearly state that the HAP-HPC/PLA nanofibrous mat of composite ratio 70:30 shows an optimal increase in tensile strength (9.53 MPa) for a decreased diameter (110 ± 66 nm).

Structurally, all molecules of the nanofibers should be fully extended and aligned perfectly with the fiber axis. The secondary bonds of the molecular structure of the fibers help in the determination of the tensile strength. The high stiffness of the nanofibers can be obtained if the polymer chains are fully extended and oriented. Some polymer chains are defective due to the high molecular weight of the polymer leading to the high tenacity of the fiber. The mechanical strength of the nanofibrous mat is influenced by the size of the fiber diameter. This confinement effect is the main property for the improvement in the mechanical strength of the mat. Hence, the orientation and degree of alignment can be influenced by decreasing the diameter of the nanofiber [32,33].

### 3.6. X-ray Photoelectron Spectroscopy (XPS)

**Figure 4:**
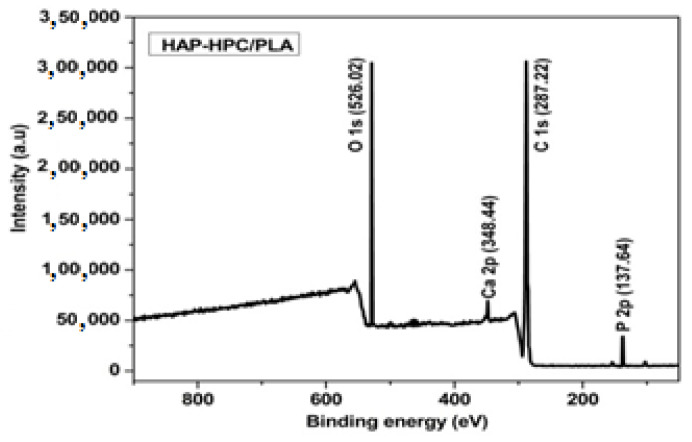

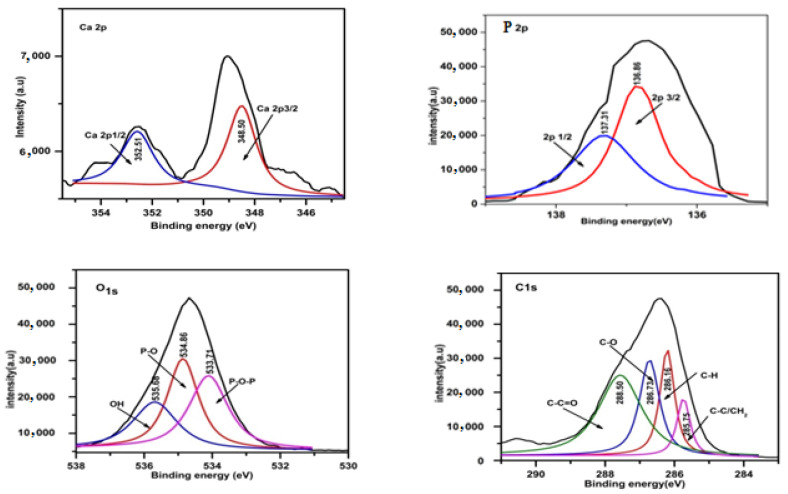
The XPS full survey for HAP-HPC/PLA nanofiber and core level spectrum of Ca 2p, P 2p, O 1s, and C1s.

The results which are obtained from FT-IR, XRD and SEM (scanning electron microscopy) analysis suggest that HAP-HPC/PLA nanofibrous mat of composite ratio 70:30 is the desired and favorable composition, and the surface chemistry of the material was analyzed using XPS. The binding energy peaks which are available in the full survey spectrum confirm the presence of Ca2p (348.44), P2p (137.64), O1s (526.02) and C1s (287.22) in the fiber matrix (Figure 4).

In HAP, the main core level of Ca (2_P_) appears at 348.44 eV region, and the two deconvoluted peaks such as Ca (2_P1/2_) and Ca (2_P3/2_) appear at 352.51 eV and 348.50 eV, respectively. Another core level of the P (2_P_) exists at 137.31.1 eV and 136.86 eV which are responsible for P (2_P1/2_) and 2(2_P3/2_), respectively [32,33]. The O1s appearing at 526.02 is deconvoluted into three elements which are present as P-O (535.68 eV), OH (534.86 eV), and P-O-P (533.71 eV), respectively. Additionally, Table 4 confirms the presence of C 1s of HPC and PLA appeared at 287.22 eV and its deconvoluted peak has four different elements at a different binding energy such as 288.50 eV (C-C=O), 286.73 eV (C-O), 286.16 eV (C-H), and 285.75 eV (C-CH_3_), respectively. Therefore, XPS analysis confirms the nanofiber matrix surface chemistry and the Ca/P (1.67) ratio, which is specific to HAP [34].

### 3.7. SEM Morphology and EDAX

**Figure 5:**
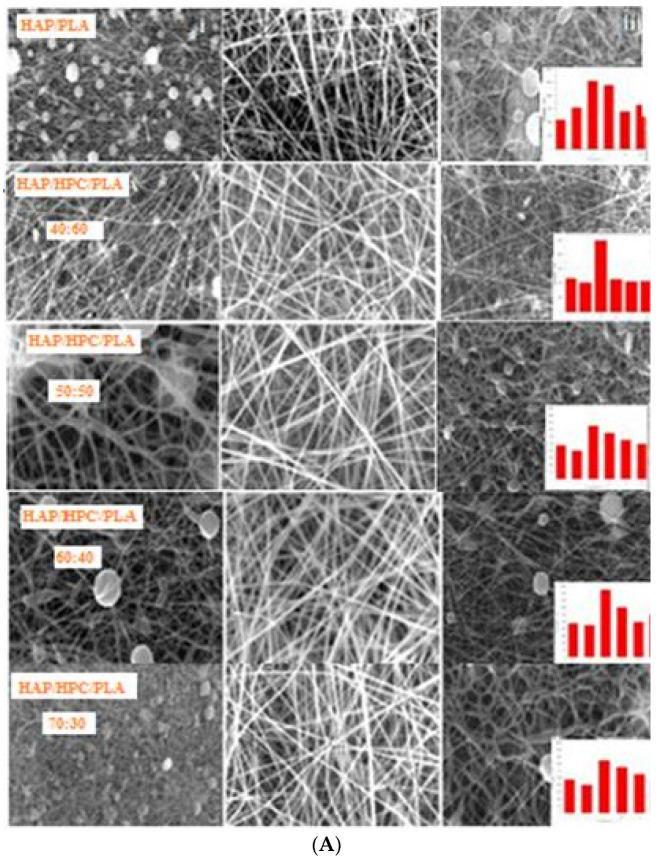

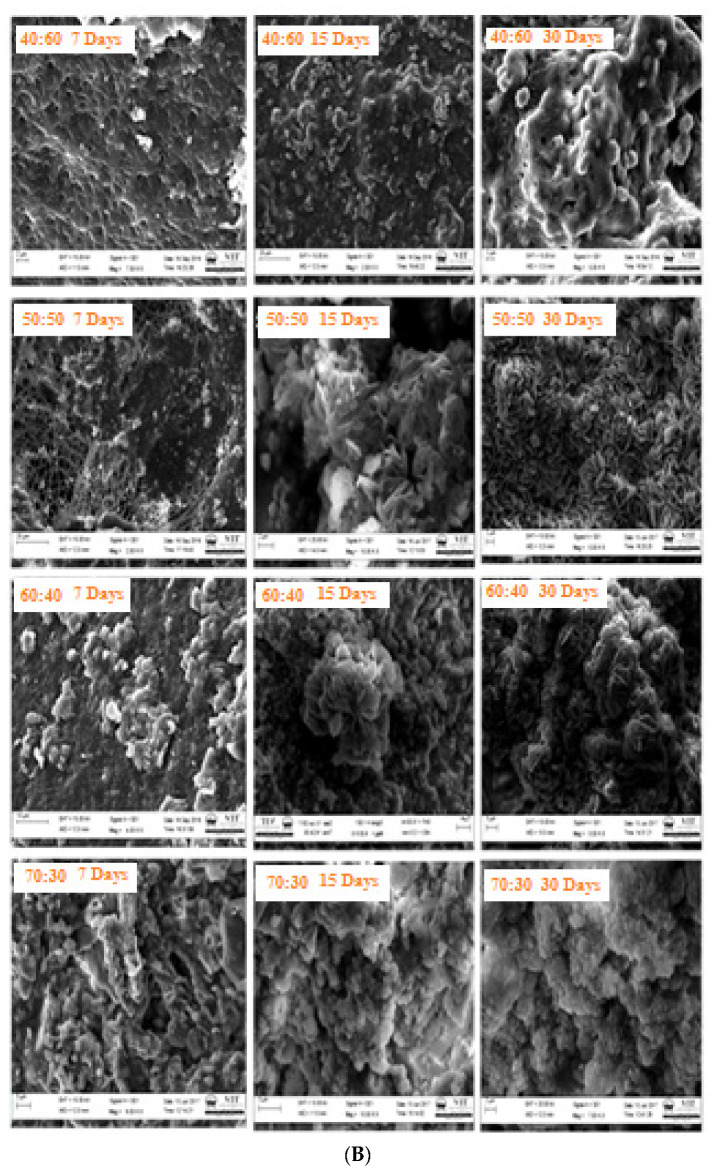
(**A**) SEM Morphology of electrospun nanofibers at various magnifications and fiber diameter (Insert) of various compositions. (**B**) SEM Morphology of SBF immersed electrospun nanofibers for various time intervals by in vitro bioactivity study.

The morphology of the fabricated HAP-HPC/PLA electrospun nanofibrous mats was analyzed using SEM. Initially, the optimization study was performed with different parameters such as flow rate (0.5, 0.7, and 1 mL), collector rotation speed (700, 1000, and 1500 rpm), tip-to-collector distance (12, 15, and 17 cm), and voltage (15, 21, and 25 Kv (kilo volts)) as shown in Table 1. Accordingly, the samples were analyzed for the morphological studies and pore size measurements using SEM and image J software, respectively (Figure 5A).

**Figure 6:**
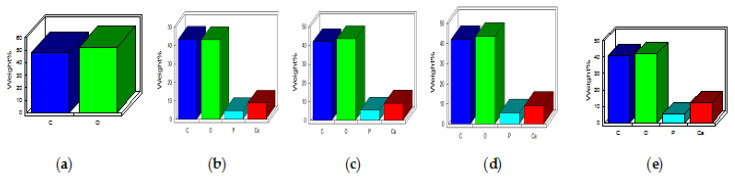
EDAX analysis of (**a**) HPC/PLA (**b**) 40:60 (**c**) 50:50 (**d**) 60:40 (**e**) 70:30 of HAP-HPC/PLA.

Figure 5A represents the SEM micrograph for HAP-HPC/PLA composite nanofibrous mat of various compositions. The optimum parameters, i.e., a flow rate of 0.7 mL, a collector rotation speed of 1000 rpm, tip-to-collector distance of 15 cm, and voltage of 21 kV gave uniform nanofibers which were bead-free, porous, and non-woven amongst all the HAP-HPC/PLA blends. The other parameters resulted in the formation of improper HAP-HPC/PLA nanofibers. The Ca and P content in the HAP-HPC/PLA nanofibrous mat were determined using EDAX (energy dispersive analysis X-ray) analysis featured in Figure 6 and Table 5 respectively. The stoichiometric value of HAP is 1.67, which is on par with the stoichiometric Ca/P (calcium/phosphate) ratio of HAP. HAP-HPC/PLA nanofibrous mat of all ratios showed the same stoichiometric ratio of Ca and P content.

The HAP-HPC/PLA mat showed different diameter sizes for each composition ratio of HAP-HPC/PLA fabrication. The higher HAP-containing mat showed a smaller fiber diameter, while the mat with no HAP showed a higher fiber diameter. From Table 6**,** it was observed that the HAP-HPC/PLA nanofibrous mat of the composite ratio 70:30 showed a diameter of 110 ± 66 nm, whereas the mat of composite ratio 40:60 showed a fiber diameter of 277 ± 11 nm. Additionally, as stated earlier, the fiber diameter decreases with an increase in the concentration of HAP. The viscoelastic property may be the cause for the reduction in diameter size with an increase in HAP concentration since all parameters remained constant.

### 3.8. Pore Size and Porosity

It has been observed that the pore size of HAP-HPC/PLA nanofibrous mats increases with increasing HAP concentration owing to a decrease in fiber diameter. The nanofibrous mat of composite ratios 0:100, 40:60, 50:50, 60:40, and 70:30 ratios yielded pore sizes of 4.54, 7.21, 9.23, 11.21, and 15.54 µm, respectively. Favoring cell proliferation and migration, pore size plays a major role in tissue engineering applications [35]. Factors such as cell growth, migration, and nutrient supply completely depend on the pore size and their interconnectivity. Cell migration becomes limited if the pore size is less, and also, decreases in the surface area may lead to limited cell adhesion if the pore size is very high. The interconnection of pores results in providing space for vasculature, required to promote new bone formation. Prior studies suggest that microporosity provides bone growth on scaffolds and it can increase the acting surface area of the mat for protein adsorption, it may also provide osteoblast attachment points during biocompatibility evaluation [36]. The pore size of the mat obtained from SEM images was processed using image J software and Porosity was measured by solvent replacement method. The results obtained from both studies are tabulated in Table 6.

### 3.9. Bioactivity

The dissolution and precipitation process assists in the formation of an apatite layer on the surface of HAP which is essential for a good calcium-based biomaterial. HAP from the nanofibrous mat releases Ca^2+^ ions when it is immersed in the SBF solution, hence increasing apatite formation in the surrounding body fluid due to this ionic activity. The release of Ca^2+^ ions may lead to an increase in positive charge on the surface of hydroxyapatite in the nanofibrous mat [37]. Further, the calcium-rich surface interacts with the PO_4_^3-^ ions present in SBF. The calcium and phosphate ions migrate onto the surface and induce precipitation of apatite on the HAP surface of the nanofibrous mat. This formed apatite gets stabilized by the crystallization process and forms a bone analog. In addition, various ions play a key role in the formation of the apatite layer due to the ionic interaction taking place between the mat’s surface and the ions present in SBF.

The apatite layer formation on the mat’s surface was shown in Figure 5B. The apatite layer formation was obtained by immersing the Mat in SBF solution at different time intervals such as 7, 15, and 30 days. The SEM images exhibit the formation of apatite layers on the nanofibrous mat’s surface as least after 7 days of immersion, and it was found that the quantity of apatite formation increases for 15 and 30 days of immersion. Further, the HAP-HPC/PLA nanofibrous mat with a composition ratio of 70:30 shows higher apatite layer formation after 30 days of immersion compared to the other composition ratios. The higher apatite layer formation is due to the high immersion time in SBF.

### 3.10. Degradation

**Figure 7:**
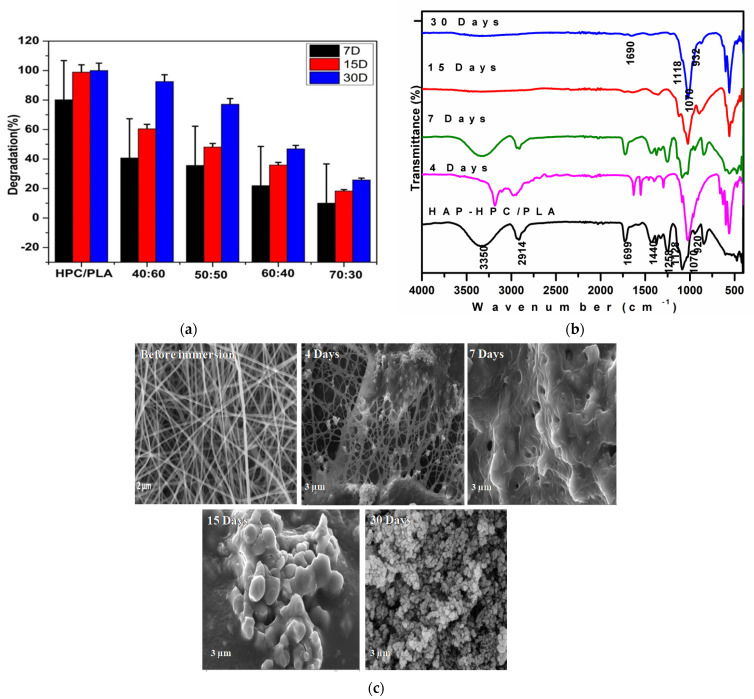
(**a**). Degradation rate of different composition ratios. (**b**). FTIR analysis of nanofibrous mat at different intervals of SBF immersion. (**c**). SEM analysis of nanofibrous mat at different intervals of SBF immersion.

The biodegradation of polymers attacks the anhydride, ester, amide groups, or even enzymatically cleaves the structural or functional bonds. The molecular weight and structure, composition, crystallinity, and presence of cross-linking in a polymer affect the polymer degradation. In addition, the degradation of a polymer is influenced by the diffusion coefficient of water in the polymer matrix, the hydrolysis rate constant of the ester bond, the diffusion coefficient of the chain fragments within the polymeric matrix, and the solubility of the degradation product [38]. The results are demonstrated in Figure 7a. The fast degradation is due to a higher concentration of polymers (40:60, 50:50) with low molecular weight and the hydrolytic reaction is high in the early stage of degradation. By increasing the concentration of HAP, the ionic exchange (ca^2+^ and PO_4_^3−^) is very high between the stimulated body fluid and nanofibrous mat when compared with a low concentration of HAP. When the ions entered into the pores, the mineralization process also occurred predominantly. Once the mineralization process occurred, the interchange of ions might be less, and the degradation was reduced.

Figure 7b, the FT-IR analysis confirms by increasing the days of immersion exhibits the polymer decrease that shows the degradation rate. After 4 days of degradation, the nanofiber breakage was obtained and there is no morphology change was observed. In 7 days of degradation, the result confirms that the surface of pore fibers was covered due to the swelling effect. In 15 and 30 days of immersion shows that interconnected fiber shape was merged together and melted during the degradation process. Figure 7c represents the SEM analysis of the SBF-immersed scaffolds at various time intervals. After 4 days of immersion, the nanofibrous scaffold starts to degrade and there was a slight change in the morphology was observed. At 7 days of degradation, the results further confirmed that the pore in the fibers was covered due to the swelling effect. At 15 and 30 days of immersion, the complete coverage of apatite was clearly visible on the surface of the scaffold. From the figure, it was clearly confirmed the process of degradation rate with respect to various time intervals. Hence from the observation, the stability of the scaffold for 4 weeks is confirmed.

### 3.11. Cytotoxic Analysis

**Figure 8:**
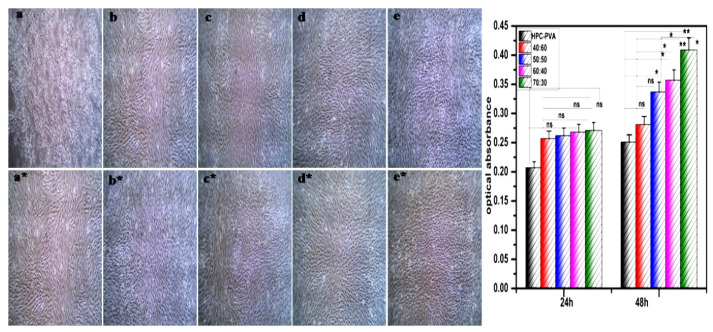
Cytotoxicity of electrospun nanofibrous (**a–e**) (24 h) and (a*–e*) (48 h) (**a**) HPC/PLA (**b**) 40:60 (**c**) 50:50 (**d**) 60:40 (**e**) 70:30 of HAP-HPC/PLA and its significant difference.

The microscopic images of the osteosarcoma (MG-63) cells seeded in HAP-HPC/PLA nanofibrous mat of different ratios (40:60, 50:50, 60:40, and 70:30) are shown in Figure 8a–e. Different patterns of cell proliferation have been observed within two days of culture. Cell proliferation differs at 24 h and 48 h, meaning cell viability is high even after 48 h when compared to 24 h. HPC/PLA nanofibrous mat shows moderate results in cell viability at 48 h (Figure 8a*) than at 24 h (Figure 8a). Moreover, these microscopic images clearly conclude that complete cell proliferation was observed after 48 h for a 70:30 composition ratio (Figure 8a*). This may be due to the infiltration of HAP with HPC/PLA composite, hence showing better performance in cell proliferation. The proliferation rate of cells in HAP-HPC/PLA nanofibrous mat is much higher at a 70:30 ratio compared to 40:60, 50:50, and 60:40 composition ratios for both 24 and 48 h cultures, respectively.

Hydrophobicity of the mat increases due to the cross-linking phenomenon and hence exhibiting cell adhesion as well as improved cellular functions. HPC and HAP usually show high biocompatibility and mechanical properties, therefore the cellulose derivatives are used as an important biomaterial for the fabrication of tissue engineering scaffolds. These biodegradable scaffolds are mainly used in tissue regeneration applications since the biodegradation rate of the scaffolds matches the biological process which takes place in tissue regeneration. Often, slow biodegradable scaffolds are preferred for regeneration applications due to minimal risks [39,40,41]. Interestingly, cellulose is an important biomaterial candidate for the design of tissue engineering scaffolds. The cells attach, grow and stimulate tissue growth depending on the stability of scaffolds in the body fluid; hence, scaffolds should be insoluble in water.

Figure 8 shows the results of the MTT assay at 24 h and 48 h at various composition ratios of 40:60, 50:50, 60:40, and 70:30 of HAP-HPC/PLA nanofibrous mat. The mat of ratio 70:30 is found to have the highest cell proliferation rate compared to other ratios at both 24 and 48 h. The electrospun nanofiber at 70:30 ratios showed a significant difference with *p* < 0.01 (Figure 8) levels in the cell proliferation for 48 h, respectively. The obtained results confirm that the cells were attached and proliferated on HAP-HPC/PLA nanofibrous mat. This may be due to the presence of β-glucose linkages in HPC. The β-glucose linkage of carbohydrate derivatives plays a major role in cell metabolism, and it activates the formation of HAP. High cell proliferation was achieved at the highest concentration of HAP in the HAP-HPC/PLA nanofibrous mat, hence proving the fact that this composition is non-toxic and significant material for regeneration and the rejuvenation of bone tissues.

### 3.12. In Vivo Study

#### 3.12.1. X-Ray Radiology Results

**Figure 9:**
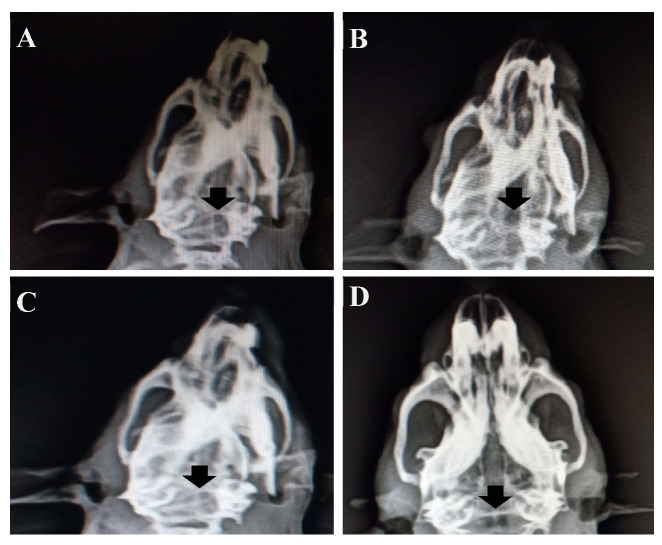
X-ray images of (**A**) without implantation at 4 weeks (**B**) HAP-HPC-PLA at 4 weeks (**C**) without implantation at 8 weeks (**D**) HAP-HPC-PLA at 8 weeks.

Figure 9 shows the X-ray examination of with and without implantation of the HAP-HPC-PLA nanofibrous composite. Instead of new bone formation, the hollow void space was observed in the defected area and is clearly visible in Figure 9A,C. Further, the results confirmed that there was no new bone formation at 4 and 8 weeks of implantation. Figure 9B displays, no osteogenesis, and bone formation were observed at 4 weeks of HAP-HPC-PLA implantation. The border of the unfilled void with no osteogenesis was observed at 8 weeks of implantation with HAP-HPC-PLA nanofibrous mat (Figure 9D). When compared with 4 weeks of implantation, 8 weeks of implantation confirmed the new bone formation. Further study is in progress to study the effect of new bone formation with respect to different ratios of HAP in the scaffolds.

#### 3.12.2. Histological Analysis

**Figure 10:**
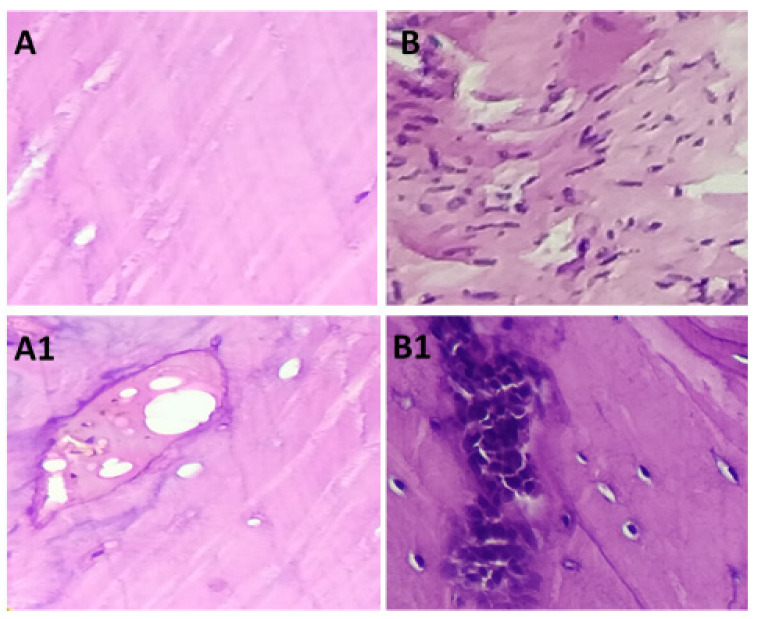
Histological images of (**A**) without implantation at 4 weeks (**B**) HAP-HPC-PLA at 4 weeks (**A1**) without implantation at 8 weeks (**B1**) HAP-HPC-PLA at 8 weeks.

Figure 10 illustrates the highlights of the study of animal experiments. The histological section of in vivo studies with and without HAP-HPC-PLA polymer composite on rat skull defected area. The results demonstrated that the scaffolds promoted bone bonding activity at 4 and 8 weeks of implantation. The results of radiology and histology analysis indicated that this scaffold facilitated bone formation in the defects with excellent potential in bone defect repair.

Figure 10B shows the development of new cells and blood vessels at 4 weeks of HAP-HPC-PLA nanofiber implantation. When compared with 4 weeks intervals, 8 weeks of implantation of HAP-HPC-PLA nanofibers showed enhanced cell proliferation such as osteoblast, osteocyte with lacunae and osteoclast (Figure 10B1). The new bone formation was started to grow at 8 weeks of implantation. Architectural modification for scaffold such as pore size and fiber diameter also seems necessary for osteoblasts to favorably attach and grow on the hybrid scaffolds to substitute collagen sponge. Such scaffolds were reported to degrade in vivo after 2–6 months after their implantation [42]. Additionally, Pektok et al. discussed the use of scaffolds with better healing properties in vivo. They showed that faster extracellular matrix formation was achieved with the decomposition of nanofibers grafts [43]. So, these nanofibers with excellent healing properties can be applied for biomedical applications. Both in vitro and in vivo studies verified that these novel layered scaffolds can effectively deliver growth factors with better cell migration in a controlled manner for bone repair by promoting the healing process.

## 4. Conclusions

Bone tissue engineering applications require fibrous mats, which have desired properties such as proper chemical integrity and crosslinking efficiency, and biodegradable properties to interact in the void space of the human native tissue without dissolving. Addressing porosity and optimum interconnectivity, optimum fiber diameter is required to ensure the necessary infiltration of cells and nutrients. The current study optimized the parameters required to produce the nanofibrous mat with the required porosity in terms of interconnectivity, mechanical property, bioactivity, and biocompatibility. Different concentrations of HAP were chosen to fabricate the nanofibrous mat resulting in the observation that the composite ratio of 70:30 exhibits all the desired properties required to be considered as an ideal biomaterial.

✓ The XRD results confirm the existence of HAP in the presence of a polymeric network and it was found that the triplet peak at 31.89°, 32.05°, and 32.20° increases with an increase in the HAP concentration in the fibrous mat.
✓ The mechanical property of 9.53 Mpa was obtained for the optimized composition with a high rate of HCA formation on SBF immersion, this may be due to the interconnected polymeric network and porosity of the sample which was confirmed favorable for cellular activity.
✓ The retention of the Ca/P ratio of HAP in the polymeric network was analyzed by XPS analysis. Finally, the biocompatibility evaluation on the MG-63 osteoblast cell line was conducted for 24 h and 48 h which deemed the material fit for biomedical applications. All the compositions revealed enhanced cell proliferation at 48 h of duration.
✓ In vivo animal study confirmed the effective bone formation at the 8th week of implantation of HAP-HPC-PLA grafted in the defective area with more cell differentiation when compared with the 4th week of implantation.

Hence, from the above study, the fabricated nanofibrous mat of this composition ratio is found to be the desirable type for bone tissue engineering applications such as bone void filling, repair of bone damage, and in vitro and in vivo bone disease modeling.

## Figures and Tables

**Table 1:** The composite mixture and the volume of the solution.

S.No.	Sample Ratio	Solution Preparation
1	HPC/PLA (0:100)	(Without HAP)10 mL of HPC/PLA
2	HAP-HPC/PLA (40:60)	4 mL of HAP and 6 mL of HPC/PLA
3	HAP-HPC/PLA (50:50)	5 mL of HAP and 5 mL of HPC/PLA
4	HAP-HPC/PLA (60:40)	6 mL of HAP and 4 mL of HPC/PLA
5	HAP-HPC/PLA (70:30)	7 mL of HAP and 3 mL of HPC/PLA

**Table 2:** Optimizing different parameters and its blending properties.

S.No.	Sample	Viscosity(cP)	Conductivity (µS/cm)	Flow Rate (1/mL)	Collector Rotation Speed (RPM)	Tip to Collector Distance (cm)	Voltage (kV)
1	HPC	63	-	0.5 0.7 1	700 1000 1500	12 15 17	15 21 25
2	PLA	4519	-	0.5 0.7 1	700 1000 1500	12 15 17	15 21 25
3	HPC/PLA	3687	0.0018	0.5 0.7 1	700 1000 1500	12 15 17	15 21 25
4	40:60	2487	0.0023	0.5 0.7 1	700 1000 1500	12 15 17	15 21 25
5	50:50	1413	0.0024	0.5 0.7 1	700 1000 1500	12 15 17	15 21 25
6	60:40	1198	0.0036	0.5 0.7 1	700 1000 1500	12 15 17	15 21 25
7	70:30	786.7	0.0041	0.5 0.7 1	700 1000 1500	12 15 17	15 21 25

**Table 3:** Mechanical properties of HAP-HPC/PLA.

S.No.	Sample	Proportional Limit (Pa)	Yield Strength (Mpa)	Tensile Strength (Mpa)	Young Modulus (Mpa)	Fracture (%)
1	HPC/PLA	0.424	0.444	3.57	131.06	148
2	40:60	0.097	0.291	4.88	180.50	111
3	50:50	0.926	4.823	8.21	190.50	219
4	60:40	1.134	2.824	8.91	370.89	118
5	70:30	1.470	1.388	9.53	430.10	90

**Table 4:** The binding energy of XPS spectra peak for HAP-HPC/PLA.

Sample	Ca 2P		P 2p		C1s	O1s	Ca/P Ratio
	2p_3/2_	2p_1/2_	2p_3/2_	2p_1/2_			
HAP-HPC/PLA	348.50	352.51	136.86	137.31	(a) 288.50-(C-C=O) (b) 286.73-(C-O) (c) 286.16-(C-H) (d) 285.75-(C-C/CH_2_)	(a) 535.68-OH (b) 534.86-P-O (c) 533.71-P-O-P	1.66

**Table 5:** The calculated Ca/P Ratio from EDAX Analysis.

S.No.	Sample	Ca/P Ratio
1	HPC/PLA	-
2	40:60	1.66
3	50:50	1.67
4	60:40	1.65
5	70:30	1.67

**Table 6:** The fiber pore size and diameter calculated from SEM images and solvent replacement method.

S.No.	Sample	Pore Size (μm)	Pore Diameter (nm)	Porosity (%)
1	HPC/PLA	4.54	355 ± 10	59.56
2	40:60 HAP-HPC/PLA	7.21	277 ± 11	62.97
3	50:50 HAP-HPC/PLA	9.23	133 ± 33	75.23
4	60:40 HAP-HPC/PLA	11.21	122 ± 33	89.45
5	70:30 HAP-HPC/PLA	15.54	110 ± 66	98.11
